# Arsenic Exposure through Dietary Intake and Associated Health Hazards in the Middle East

**DOI:** 10.3390/nu14102136

**Published:** 2022-05-20

**Authors:** Mohammad Idreesh Khan, Md Faruque Ahmad, Irfan Ahmad, Fauzia Ashfaq, Shadma Wahab, Abdulrahman A. Alsayegh, Sachil Kumar, Khalid Rehman Hakeem

**Affiliations:** 1 Department of Clinical Nutrition, College of Applied Health Sciences in Arras, Qassim University, Buraydah 58883, Saudi Arabia; moi.khan@qu.edu.sa; 2 Department of Clinical Nutrition, College of Applied Medical Sciences, Jazan University, Jazan 45142, Saudi Arabia; farooqui@jazanu.edu.sa (F.A.); aalsayegh@jazanu.edu.sa (A.A.A.); 3 Department of Clinical Laboratory Sciences, College of Applied Medical Sciences, King Khalid University, Abha 62529, Saudi Arabia; irfancsmmu@gmail.com; 4 Department of Pharmacognosy, College of Pharmacy, King Khalid University, Abha 61421, Saudi Arabia; 5 Department of Forensic Chemistry, College of Forensic Sciences, Naif Arab University for Security Sciences (NAUSS), Riyadh 14812, Saudi Arabia; sachilvohra@gmail.com; 6 Department of Biological Sciences, King Abdulaziz University, Jeddah 21589, Saudi Arabia; khakim@kau.edu.sa; 7 Princess Dr. Najla Bint Saud Al- Saud Center for Excellence Research in Biotechnology, King Abdulaziz University, Jeddah 21589, Saudi Arabia; 8 Department of Public Health, Daffodil International University, Dhaka 1207, Bangladesh

**Keywords:** arsenic, Middle East, health hazards, water, food and screening

## Abstract

Dietary arsenic (As) contamination is a major public health issue. In the Middle East, the food supply relies primarily on the import of food commodities. Among different age groups the main source of As exposure is grains and grain-based food products, particularly rice and rice-based dietary products. Rice and rice products are a rich source of core macronutrients and act as a chief energy source across the world. The rate of rice consumption ranges from 250 to 650 g per day per person in South East Asian countries. The source of carbohydrates through rice is one of the leading causes of human As exposure. The Gulf population consumes primarily rice and ready-to-eat cereals as a large proportion of their meals. Exposure to arsenic leads to an increased risk of non-communicable diseases such as dysbiosis, obesity, metabolic syndrome, diabetes, chronic kidney disease, chronic heart disease, cancer, and maternal and fetal complications. The impact of arsenic-containing food items and their exposure on health outcomes are different among different age groups. In the Middle East countries, neurological deficit disorder (NDD) and autism spectrum disorder (ASD) cases are alarming issues. Arsenic exposure might be a causative factor that should be assessed by screening the population and regulatory bodies rechecking the limits of As among all age groups. Our goals for this review are to outline the source and distribution of arsenic in various foods and water and summarize the health complications linked with arsenic toxicity along with identified modifiers that add heterogeneity in biological responses and suggest improvements for multi-disciplinary interventions to minimize the global influence of arsenic. The development and validation of diverse analytical techniques to evaluate the toxic levels of different As contaminants in our food products is the need of the hour. Furthermore, standard parameters and guidelines for As-containing foods should be developed and implemented.

## 1. Introduction

As is a naturally found crystalline metalloid with ubiquitous distribution throughout the earth’s crust. As exposure to the human food chain ecosystem comprises air, water, food, and soil. Daily diet contamination depends on the inorganic or organic forms, oxidation state, water solubility, and food matrix. The differentiation and categorization of different foods as sources of inorganic and organic As contamination in daily life is an important issue. Industrialization, urbanization, and anthropogenic activities such as glassware, industrial chemicals, lead alloys, and pharmaceuticals manufacturing processes are prime causes of arsenic exposure in the environment. Heavy metal concentrations in all-natural water reservoirs exceed the cut-off of WHO’s safe limits for human health [1,2].

It is found that both organic and inorganic forms have no taste, odor, or color. Pentavalent arsenic (As (V) or arsenate) and trivalent arsenic (As (III) or arsenite), both in inorganic and organic forms, are present in natural ecosystems [3,4,5]. Inorganic arsenic (As (III) and As (V) or a combination of both) in ground water and biological activity transformation converts into arsenobetaine and different arsenosugars. Moreover, lipid-soluble arsenic forms, namely arsenolipids, have also been detected in fish and algae. Different As species can be seen in Table 1.

The demand for food supply and agricultural growth leads to the use of arsenic in various fertilizers and insecticides, which are aggravating factors of heavy metals in soils and crops, including calcium arsenite and copper acetoarsenite (Paris green) as pesticides and other herbicides such as methylarsenic acid and dimethylarsenic acid [15,16]. Staple food, beverage, and seafood consumption are increasing in day-to-day life. Only a few studies have correlated the relationship between diet and As concentration and some directly measured water arsenic intake [3,17]. In the Bangladeshi population, it has been reported that two food groups, namely cereals (53–60%) and vegetables (25%), contribute to inorganic arsenic (iAs) exposure [18]. Similarly, 54–85% of total arsenic exposure is found due to rice and other foods in the United States [19]. As per physiological and nutrition requirements, children and adolescents are at more risk of arsenic toxicities due to high energy and fluid allowances per body weight [20,21]. As in infants’ cereal and baby food (USA) gains too much attention because there are no specific regulations or guidelines, but the safe limit is 0.2 mg/kg for infants and young children [22,23]. According to the WHO, arsenic is among the most deadly substances impacting human wellbeing and is highly poisonous in its inorganic form. Long-term exposure increases the risk of cancer, skin lesions, pigmentation, cardiovascular disease, and diabetes. In utero and early childhood exposure to arsenic leads to poor cognitive function and increases teenage mortality [24,25,26,27].

## 2. Facts of Arsenic Exposure

### 2.1. Arsenic Ingestion through Water Ecosystem

The biggest hazard to the health care system from arsenic is groundwater contamination. As mobilization in groundwater is related to geologic setting and sedimentary components that regulate the geochemistry and As release from bedrocks into groundwater [28,29]. Mahmoud et al. (2018) assessed the quality (physicochemical parameters and heavy metals) of household drinking water gathered at four sampling points in Abu Dhabi’s Baniyas district and the United Arab Emirates [29]. Primary research revealed that several households had amounts of As, Cd, and Pb that exceeded the overall permissible limit set by UAE drinking water guidelines. Furthermore, as opposed to the other sampling points, the main water supply, the samples had a higher heavy metals concentration. Overall, the principal components analysis (PCA) showed that certain physical parameters could have contributed to high heavy metal levels. In a few samples of Kuwait marine water and sediments, it was observed that most heavy metals such as Cr, As, Ni, Hg, and Zn were within toxic limits with the exception of Cd, Cu, and Pb [30]. Okati et al. (2021) evaluated Hg, As, and Se levels in six Oman sea fish species and assessed the health risks of Hg and As toxicity, concluding that the consumption of such fish is not at high risk of As exposure [31]. Sabarathinam et al. (2019) compared Kuwait Bay seawater and revealed that the majority of heavy metals such as B, Li, Hg, Sr, Pb, Ba, Zn, Fe, Be, Mn, Co, Cd, Cr, Se, Ni, Al, V, Mo and As were present in higher concentrations [32].

Molamohyeddin et al. (2017) investigated the content of heavy metals in the surface sediments of Chabahar Bay and Oman. At certain stations, the arsenic level was higher than the effect range low (ERL) [33]. Statistical studies showed that organic matter and mud play an important part in metal dispersion. Similarly, Agah et al. (2016) found that the metal contents of the sediments was in the order listed: Al > Fe > Cr > V > Ni > Zn > Cu > As > Pb > Co [33]. As values of greater amounts were observed in certain regions, reflecting anthropogenic inputs. In another study, AI-Kalbani et al. (2001) evaluated the water quality of nine Aflaj samples in Al Jabal Al Akhdar, Oman [34]. Mg, Mb, and As concentrations in some Aflaj water samples were only just over the cap. Al-Farraj et al. (2013) evaluated the distribution of As and related hydrogeochemical parameters in 27 randomized aquifer-based boreholes in Saudi Arabia’s AI-Kharj agricultural area [35]. The contents of As were detected in every area with 92.5 percent of boreholes yielding concentrations that were greater than the WHO’s allowable maximum concentration of 10 μg/L. The maximum concentration was calculated to be 122 μg/L. Long-term exposure to high levels of iAs in drinking water has been linked with skin disorders and increased risk of diabetes, high blood pressure, and several types of cancer. iAs and As compounds are considered to be carcinogenic [36,37].

Ghrefat et al. (2016) analyzed the overall abundance and spatial distribution of many metals in the groundwater and farmland soils in Saudi Arabia’s Gulf of Aqaba (GoA) [38]. The metal amounts analyzed in the groundwater samples were below the WHO’s permissible limits. Similarly, Bu-Olayan et al. (2001) observed low As levels in the majority of marine organisms off the coast of Kuwait [39]. Further, in other studies, the quantities and concentrations of trace metals on the surface of seawater along the eastern coast of Saudi Arabia were investigated. It was observed that the concentrations of Zn, Fe, Cu, Se, and B were decreasing northward. Although As, Cd, Mn, and Pb levels were increased in the northern GoA. The amount of metal in the water was determined by nearby geologic materials [40]. In other studies, seawater was collected from 23 separate locations identified as fishing areas of the Kingdom of Bahrain’s territorial water for the purpose of creating a benchmark and evaluating marine pollution due to heavy metals [40]. The findings indicated that the coastal waters seem to be of good quality as the concentrations of metals recorded were much lower than those of the United Kingdom water quality standards, except for Cu at all sites and Hg at the Masoor site. De Mora et al. (2004) assessed heavy metal contaminants in the Gulf and Gulf of Oman using aquatic biota (fish and various bivalves) and coastal sediment collected in Bahrain, Oman, Qatar, and the UAE. Sediment metal loadings were typically not noteworthy, though hotspots of different heavy metals were noted in Bahrain (Hg, Cu, Pb, Zn) and UAE’s east coast (Co, As, Ni, Cr). As and Hg concentrations in sediments were usually low and overall Hg levels in top predator fish widely eaten in the area were 0.5 μg/g and posed no hazard to human health. Certain bivalve species from the area had extremely high As concentrations (up to 156 μg/g), though it cannot be confirmed that As is more likely produced from natural causes rather than anthropogenic pollution [41]. Moreover, Al-Awadi et al. (2003) found that As concentrations in the water samples from all 42 wells were lower than the detection limit of 0.005 mg/L. Various abnormal concentrations of As can be seen in different coastal areas of diverse countries in Table 2.

### 2.2. Arsenic Ingestion through Dietary Contaminant Food

The dietary and food contamination of iAs is a matter of concern due to its carcinogenic potential. Children’s dietary components are mainly based on a staple diet, rice and infants’ formulas, with high iAs content. Gu et al. (2020) reported that 75% of rice-based infant food from Australia contains high iAs (0.1 mg/kg) due to rice and whole-grain mix cereals [48]. A similar finding was noticed by Hernández-Martínez and Navarro-Blasco (2013) in Spain and Juskelis et al. (2013) in American infants’ cereals. Another study carried out in Ireland and the UK reveals that formula-fed infants had higher concentrations of urinary dimethylamine (DMA) and methylmalonic acid (MMA) than exclusively or partially breastfed infants due to high exposure [49,50]. These As metabolites in post-weaning infants were found due to a complementary food formula based on rice cereals that were higher than the EU limit of 0.1 mg/kg [10]. Middle East regions, especially Gulf countries, have diets mainly consisting of rice-based foods. In another review, infants’ cereals in the Middle Eastern market showed exponential growth due to some social factors such as ready-made cereals, ready-to-eat restaurant practices, food processing technology, and the use of different additives in manufacturing processes, which, in Middle Eastern countries, are the main cause of dietary heavy metals contamination. The total iAs contamination of daily-use food can be seen in Table 3.

As-rich soil, water, geochemical activity, and pesticides lead to the contamination of the food chain. Arsenic exposure in nearly 1 billion of the population is through food and >200 million of the population through drinking water beyond the exposure limit of 10 μg/L [70]. As toxicities through food depend on food choice, culture, age group, and dietary restrictions. The consumption of a single food throughout the year without seasonal variety might be a worsening factor of toxic exposure. The ingestion of As through rice and legumes is considered a public health problem [3]. Rice has the highest capacity to absorb arsenic as compared to other cereal grains such as barley, wheat, oat, rye, and corn. Risk assessment was analyzed by the EFSA [71]. The European population concluded that cereals and processed foods are the primary cause of iAs exposure in the population, while water, rice, and dairy products exhibit a major role in iAs exposure in infants and toddlers. Other seafood and beverages such as apple juice have also been considered as a source of iAs toxic exposure. Lynch et al. (2014) estimated mean values in four different food groups, including seaweed/algae at 11,000 μg/kg, seafood at 130 μg/kg, rice at 130 μg/kg, and other cereal-related products at 92 μg/kg [72]. Arsenate (iAsV) or arsenite (iAsIII) are inorganic forms of arsenic present in food and drinking water—after ingestion, iAsV is converted to iAsIII—whereas seafood contains organic forms of arsenic, arsenobetaine and arsenocholine [7]. Root vegetables accumulate the highest As content and the lowest edible parts [73]. As contamination in various types of food and beverages in combination plays a major role in the progression of dangerous diseases such as cancer. Cancer has turned out to be an alarming public health concern for the whole world [74,75,76,77]. Consequently, the EFSA and JECFA reported a benchmark dose level (BMDL): 0.3–8 mg/kg body weight per day can be the cause of various kinds of cancer such as lung, skin, and bladder cancers.

## 3. Arsenic Cellular Metabolism

iAs present in the human body is generally excreted through urine and bile [78]. Urinary arsenic measurements (iAs%, MMA%, and DMA%) act as indicators of arsenic metabolism and methylation capacity [79,80]. Trans-cellular and paracellular pathways are major modes of iAs transportation [81]. Cellular metabolism includes methylation in four forms, namely monomethylarsonic acid (MMA^V^), monomethylarsonous acid (MMA^III^), dimethylarsinic acid (DMA^V^), and dimethylarsinous acid (DMA^III^). Among the different forms, MMA^III^ is reported as the most cytotoxic [82]. MMA^III^ species can inhibit mitochondrial I and III processes by electron escape through the electron transport chain, leading to the production of reactive oxygen species (ROS) and reactive nitrogen (RNS). Free radical production leads to DNA damage and impaired gene expression [83]. Enzymatic methylation occurs by way of the primary enzyme involved in As metabolism, called arsenic (3+) methyltransferase (AS3MT), and endogenous reducing agents such as thioredoxin (Trx) and glutathione (GSH) [84,85].

Arsenic has an affinity with thiol groups, inhibiting the catalytic activity of an enzyme by binding with thiol-containing active sites. GSH plays an important role in transforming arsenate (AsV) to arsenite (AsIII); the arsenite form has a shorter half-life in comparison to arsenate. Antioxidants act as electron donors during the reduction of pentavalent to trivalent arsenic due to their high affinity to GSH [86]. Arsenic–thiol interaction consequences include MMA^III^ inhibiting GSH reductase and thioredoxin reductase [87]. Arsenate produces glucose-6-arsenate and 6-arsenogluconate by the substitution of phosphate in glucose and gluconate and forms glucose-6-arsenate and 6-arsenogluconate, analogous to glucose-6-phosphate and 6-phospho-gluconate, respectively. Glucose-6-arsenate binds to glucose-6-phosphate dehydrogenase and a high concentration of arsenate inhibits hexokinase activity through negative feedback mechanisms during glycolysis. Arsenic inhibits the conversion of pyruvate to acetyl coenzyme A (acetyl-coA), which leads to diminished cellular glucose uptake, gluconeogenesis, the oxidation of fatty acid, and further acetyl-CoA production. Mitochondria is an important cellular target by arsenite and free radical production, lipid peroxidation, H_2_O_2_ production, and mitochondrial swelling. Arsenic induces the formation of superoxide anion radicals such as singlet oxygen, the peroxyl radical, hydroxyl radicals, NO, H_2_O_2_, dimethyl-arsinic-peroxyl radicals, and dimethylarsinic radicals in a dose-dependent manner and consequently leads to health complications [88].

## 4. Arsenic-induced Health Hazards

### 4.1. Major Organ Damage and Chronic Disease Development

Human body organs are typically distressed by As poisoning. Major organs susceptible to As toxicity are the kidneys, lungs, liver, and skin. Severe As toxicity leads to coma and death. Target organ damage (TOD) depends on the ingested arsenite (As^+3^) content in the body. Firstly, an initial sign of skin changes involves its binding with keratin and accumulation in hair and nails. The appearance of keratosis is a common early sign of arsenic exposure. Recently, squamous cell carcinoma (SQCC), melanosis, and keratosis to Bowen’s disease have been reported in Asian countries, especially in India, Nepal, and Bangladesh [89,90]. Increased monomethylarsonate (MMA)% and decreased dimethylarsinate (DMA)% of arsenic species are directly proportional to a higher risk of bladder, pulmonary, and skin cancers. Carcinogenic and chromatin alteration have been exhibited due to transcription initiation and gene sequencing. The cellular genomic modification of deoxyribonucleic acid (DNA) methylation and the post-transcriptional modification (PTMs) of histone proteins lead to tumors and benign dysplasia. Arsenic induces miRNA gene expression that affects polymerase elongation and the recruitment of splicing regulatory factors and leads to carcinogenicity [8,91].

Drinking water iAs is nearly 80–90% absorbed by the intestine and protein transporters of arsenic such as aquaporin-10, GLUT-5, and organic anion-transporting polypeptides (OATPB) in the gut epithelium [92]. As exposure can lead to developing a risk of nonalcoholic fatty liver diseases among adolescents [93]. As is a promoter of inflammation, oxidative stress, and endothelial dysfunction by different mechanisms including the activation of transcription factors such as protein-1 and nuclear factor κβ [94,95]. The mechanisms of carcinogenesis take place through multiple pathways, including the perturbation of gut microbiota, genotoxicity, and epigenetic dysregulation [96,97,98]. The conjugation of arsenic with glutathione forms arsenic triglutathione, and the methylation of arsenic produces dimethylarsenic glutathione and enters bile and the bloodstream. This unstable species changes into the volatile compound dimethylarsine. As is transported via RBCs and is stored as protein-bound trivalent dimethyl arsenicals in different organs of the body [7]. As exhibits carcinogenic effects in the liver and produces hepatocellular carcinoma through DNA repair inhibition and the development of micronuclei and epigenetic dysregulation [96,99]. Different As species-linked health effects have been mentioned in Table 4.

Chronic exposure to As produces harmful effects on the immune system. Immune responses depend on the proliferation of T and B cells as well as macrophages. Chronic exposure to As may cause immunosuppression by affecting cellular and humoral immunity [102,117]. It has been found that immunosuppression due to decreased T-cell proliferation is linked with low cytokine secretion, tumor necrosis factor (TNF)-α, interferon-γ, IL-2, IL-10, IL-5, and IL-4. A similar study states that chronic exposure leads to high serum immunoglobulin IgA, IgG, and IgE, which may lead to the development of respiratory complications such as pneumonia, allergic bronchitis, and chronic obstructive pulmonary disease [118]. Chronic exposure to As leads also to several other clinical manifestations such as hypercalciuria, glomerulonephritis, acute tubular necrosis, albuminuria, nephrocalcinosis, and renal papillae necrosis [81]. The development of incipient nephropathy and the incidence of chronic kidney disease (CKD) have been reported due to As-induced injury of the nephron [119]. As nephritis and renal damage occur due to direct podocyte injury and endothelial dysfunction and increase the expression of the vascular cell adhesion molecule 1 (VCAM-1) [120,121]. The direct effects of arsenic species and metabolites on chronic diseases with their respective mechanisms are listed in Table 5.

As affects the gluconeogenesis in muscle cells by inhibiting glucose transporters and suppressing glucose metabolism regulatory genes. Hence, the glycolytic pathway and mitochondrial energy production are altered [132,133]. In experimental studies of mice, it has been reported that As decreases the functional capacity of muscle and destroys muscle progenitor cells [134]. Muscle damage occurs by inhibiting muscle repair and increasing the nuclear factor kappa light-chain enhancer of activated B cells (NF-κB), along with inflammation signaling, a long healing time, and fibrosis. Consequently, As exposure contributes to sarcopenia progression [135].

**Figure 1:**
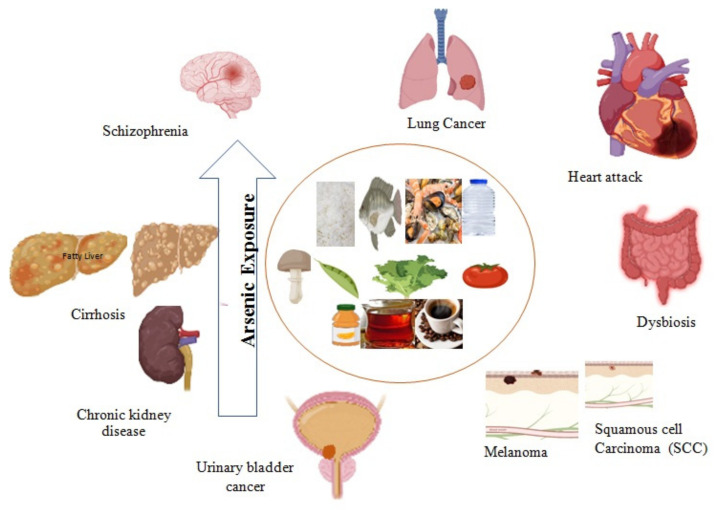
Arsenic exposure leads to acute to chronic complications such as chronic kidney disease, cardiovascular disease, schizophrenia, fatty liver, cirrhosis, dysbiosis, and different types of cancer.

As acute toxicity generates oxidative stress and pro-inflammatory reactions in the epithelial cells of the intestine in in vivo studies [136,137]. Therefore, reactive oxygen species (ROS) damage the cytoskeleton and cause the loss of tight junction proteins such as claudin-5 and occludin in the blood–brain barrier [138]. iAs species might be liable to increase paracellular transport at tight junctions [139]. The high permeability of intestinal junctions is related to intestinal abnormalities such as dysbiosis, colitis, Crohn’s disease, ulcerative colitis, and other gastrointestinal complications [140]. High As levels increase colonies of pathogenic bacteria in dysbiosis, whereas low levels directly increase intestinal commensal bacteria. Therefore, gut microbiome health depends on a variety of species of probiotic bacteria [141]. A study conducted on children exposed to high As shows plenty of proteobacteria in stool samples [142]. A recent study on the Bangladeshi population demonstrated the toxic effect of high As on the species and flora of gut bacteria and the high number of pathogens [143]. Furthermore, it has also been seen that different forms of As species cross the blood–brain barrier, reducing neurotransmitters, including mono-amines and those associated with the cholinergic, dopaminergic, and glutamatergic systems, leading to damaged synaptic transmission [144]. Glutamate receptor expression inhibition may cause changes in synaptic plasticity, for instance, the long-term potentiation of learning and memory linked with enhancing extracellular glutamate levels [145]. Various diseases linked with As toxicity are depicted in Figure 1.

### 4.2. Effects on Maternal Health

A recent study of As exposure and gestational diabetes mellitus (GDM) showed their strong positive links to each other [146]. Sung et al. reported a negative metabolic effect of As toxicity in GDM [126]. iAs changes glucose homeostasis by switching phosphates in adenosine triphosphate (ATP) synthesis and impairing ATP-dependent insulin. Furthermore, arsenate conjugates with the disulfide bridges of insulin, insulin receptors, glucose transporters (GLUTs), and glucose metabolism enzymes. Peroxisome proliferator-activated receptor γ (PPARγ) plays a significant role in the expression of insulin activation. As free radical damage interferes with the signal transduction and gene expression of β-cells, leading to the development of diabetes. As activates superoxide and binds with uncoupling protein 2 (UCP2), consequently decreasing insulin secretion [147]. UCP2 acts as a negative regulator for the secretion of insulin and also mediates proton leakage across the inner mitochondrial membrane [148].

In a research study on high exposure to As in Bangladeshi women, it was concluded that As exposure is the cause of cervical cancer (squamous cell carcinoma) [149]. Further, in another study of Bangladeshi women, it was found that As is also responsible for anemia. A prevalence of anemia has been found in the reproductive female age group and was linked to arsenicosis skin lesions [150]. High exposure to As also leads to early menopause of two years compared to low- or normal-limit exposed females’ menopausal stages [151]. In vivo and in vitro research of iAs exposure has revealed that As can attach to human and animal hemoglobin and can alter morphology, cell shape, levels of hemoglobin, and heme metabolism. Absorbed arsenic is transported through portal circulation via RBCs and WBCs. In another study, a 2–3-fold higher prevalence of anemia has been reported with a low dose of As-exposed drinking water compared to normal potable water, and pregnant women were more susceptible than non-pregnant women [152,153].

Ahmed et al. reported the effect on cellular innate immunity and immunosuppression of prenatal As exposure in Bangladeshi women [154]. The immune-suppressive effect on T and B cells as well as macrophages is due to the reduced expression of major histocompatibility complex (MHC) class II molecules, CD69, interleukin-1 beta (IL-1β), and tumor necrosis factor-alpha (TNF-α). Inflammatory cascades of cytokine release low lymphocyte proliferation and IL-2 secretion, leading to inflammation, macrophage adhesion, phagocytosis, the increased apoptosis of peripheral blood mononuclear cells (PBMC), reduced ROS stimulus by PBMC, and stop the progress of the immunogenic response in hosts [102]. The disruption of estrogen receptors and the suppression of the signaling pathway of estrogen is linked to breast cancer, and As is a potential metallo-estrogen and acts as a medium to promote breast cancer [155,156,157]. Marciniak et al. (2020) reported in a Poland study that high-dose arsenic exposure increases the risk of breast cancer by 13 times compared to normal women [158].

### 4.3. Effects on Fetal and Neonatal Health

As exposure’s adverse effects have been reported also in neonatal health. It has been observed that arsenic exposure during the last trimester of pregnancy directly affects newborn telomere length (TL). Telomeres are DNA–protein structures; they are present at the end of each strand of DNA and defend the genome from nucleolytic degradation, interchromosomal fusion, and unnecessary recombination. Thus, telomeres exhibit a significant role to preserve information within the genome. Prenatal arsenic toxicity is the cause of newborn telomerase elongation and may suggest a new approach to neonatal health hazards [159]. Exposure to arsenic has harmful genotoxicity in newborns, DNA strand breaks, and increased MN frequency in cord blood. Increased arsenic maternal biomarkers are associated with genetic defects in newborns. Milton et al. (2017) reported the effect of As on spontaneous abortion and stillbirth, which are increased by up to 2–3-fold, and the risk of complications is 6-fold higher than in unexposed women. The ingestion of high arsenic content in food and water reduces methylation, which leads to folate deficiency and high homocysteine in urine, significantly contributing to congenital malformations and placental abruption [160,161,162].

**Figure 2:**
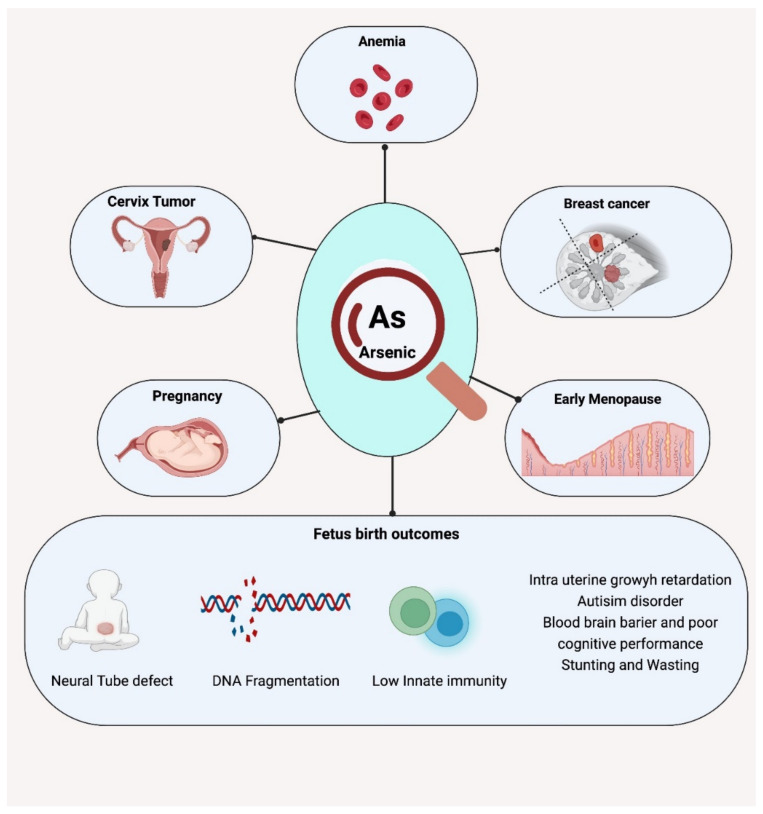
Arsenic-induced maternal health complications such as anemia, early menopause, cervical and breast cancer, and child health complications including neural tube defects, DNA fragmentation, intrauterine growth retardation, and autism.

Neural tube defects (NTDs) and insufficient neuron growth, as well as self-regulation in newborns, have been reported due to maternal As exposure [163,164]. Similar effects on newborns with high arsenic exposure were also reported in a Turkish study [165]. Deficits in memory, attention, and IQ from early life exposures are also noted with exposure to As [166]. Quansah et al. (2015) analyzed prospective birth cohort study results that indicate increased risks of spontaneous abortion, stillbirth, and neonatal and infant mortality among populations highly exposed to arsenic in drinking water. Chronic iAs exposure leads to placental insufficiency complications including preterm delivery and intrauterine growth retardation (IUGR) [154]. During pregnancy, As inorganic forms and their methylated metabolites cross the placenta and enter cord blood, leading to altered immune cell and gene expression in the cord blood of a highly exposed mother [167,168,169,170]. Moreover, other fetal complications also reported include slow fetal growth, low birth weight, and the effect of neuronal development in early life [89,171]. Many case-control and observation studies on As exposure have shown that it delays cognitive function and causes low intelligence quotients [172,173]. Children up to five years of age are more susceptible to arsenic exposure due to the high consumption of baby foods and more demand for energy and carbohydrate-rich diets. As-associated maternal and fetal complications can be seen in Figure 2.

## 5. Arsenic Screening

The most consistent method to examine arsenic exposure is with a urine test. Urine and blood tissue are convenient screening methods for the detection of heavy metals. More than 90% of arsenic exposure is detected through urine samples. The accumulation of As in hair and finger or toenails is considered chronic exposure due to the binding of sulfhydryl groups of keratin found in hair and nails and deposition takes approximately 2 weeks [173]. Hair and nails grow slowly due to multiple phages and the toxic accumulation of heavy metals, and toxic accumulation takes more than 3–6 months [174]. Sample hair and nails quantify the chronic exposure of iAs, while blood and urine measurements indicate a short duration of exposure because the half-life of arsenic in blood is 2–6 h and 4 days in urine [47,119,120]. Wang et al. (2017) examined a salivary screening detection method. Saliva collection is easy and convenient for all age groups and is found to be appropriate for children and menstruating women [79].

There are different toxic levels of iAs that have been identified in various samples such as scalp hair arsenic (1.0 < 3.0 mg/kg), toenail arsenic (>0.5 μg/g), total organic blood arsenic (>130 nmol/L), urinary arsenic (>100 μg/L), and spot urine sample arsenic (>50 μg/L), considered as upper abnormal limits [175]. An oral intake of 100–300 mg (1–5 mg/kg BW) of iAs in humans usually leads to death within 1 h, if untreated [175,176].

## 6. Food Safety and Policy Interventions

Rice is a major part of the Saudi diet but is not domestically produced. It is estimated that 1.45 million metric tons (MMT) of rice were consumed in the kingdom of Saudi Arabia (KSA) in 2015–2016 [177]. The maximum allowable concentrations of iAs, as per the European Commission regulations (European Commission, 2015), are 0.25 and 0.20 mg/kg for brown and white rice, respectively. Several Asian countries such as India and Bangladesh have not set any limits or regulations so far. Recently, around half of the rice brands in the UK have potentially exceeded the hazardous limits and are unsafe for infants and young children [178]. The arsenic content of rice varies depending on geographic and water sources. Paddies cultivated in the United States (US) have higher total As (tAs) and low iAs arsenic as compared to Indian and Bangladesh rice cultivations [179]. The recommendation of polished rice instead of brown rice and basmati rice varieties is an alternate approach to counter the risk of iAs exposure through rice [180,181]. The scientific cooking method of parboiled and absorbed (PBA) rice was investigated for the removal of 54% and 73% of iAs from brown and white rice, respectively; therefore, meal preparation is another safe approach for iAs exposure [178,182]. Dehusking, milling, parboiling, and cooking methods for raw and cooked rice are important steps to minimize As content. Meal preparation techniques, washing and rinsing time, and the temperature of boiling water determine the concentrations of arsenic species transformations [183]. Inorganic forms, arsenite (As^3+)^ and arsenate (As^5+^) are the predominant forms of As present in rice and its products [23].

An effective mitigation strategy to minimize the soluble arsenite forms in whole grain/polished rice is by cooking with a high volume of water [184]. Practicing a traditional method of cooking involves washing several times and then cooking in a large volume of water followed by draining the extra water once the rice is cooked. When the rice is boiled using sufficient water where no extra water is left after the rice is cooked results in the presence of high As levels in cooked rice. Raab et al. determined tAs and iAs content in a variety of basmati, long grain, polished, and whole rice by washing, rinsing, and boiling/steaming methods in low-volume (rice-to-water ratio 1:2.5) and high-volume (ratio of water to rice: 1:6) uncontaminated water [185]. Findings suggested that a high volume of water during preparation reduces both tAs and iAs by 35% and 45%, respectively, in long-grain basmati rice as compared to raw rice [185]. Recent studies suggested that washing three times with deionized water reduces tAs content in both white as well as brown rice by up to 81–84% and 71–83%. A similar finding was documented by rinsing with a 10:1 water:rice ratio with deionized water, reducing iAs in all varieties of brown rice [186]. Continual-stream percolating water reduces As content in cooked rice. The percolation cooking method uses a continual stream of percolating water through a filter unit by a coffee maker machine, wherein the rice is placed in the filter unit instead of coffee and boiling water is passed through the filter unit continuously. A reduction in iAs has been reported in raw polished rice by 59% and wholegrain rice by 69%, respectively [187]. Various factors such as high washing, soaking, and cooking rice with arsenic-contaminated water affect the final dietary arsenic ingestion in meal preparation [188].

Critical control points during the cooking of rice and other grains in different settings and standard protocols should be developed. The awareness of adopting proper safe cooking measures should be highlighted among the community and culinary workers. The safe upper limit of As content on labeling might be helpful for consumers of imported rice and other grains in international food markets and especially in Middle Eastern countries. Different countries’ arsenic levels in rice and other foods are listed in Table 6.

WHO Standards for Drinking Water Safety (1996) defined a tentative value of 0.01 mg/L for As in drinking water. A majority of European countries have implemented the WHO interim recommendation of 0.01 mg/L as their standard. In 1996, Australia introduced an even higher standard for As in drinking water of 0.007 mg/L [192]. The EPA interim maximum contaminant level for As in drinking water was 0.05 mg/L in the United States, but the EPA adopted a revised limit of 0.01 mg/L in drinking water in 2001. Since 2006, all drinking water delivery agencies in the United States have been adhering to the new 0.01 mg/L requirements. China, India, and Bangladesh are among the nations where the national limit for As in drinking water is 0.05 mg/L. Just about all nations in the Eastern Mediterranean Region lack a consistent plan for establishing, enforcing, and reviewing drinking water quality requirements. The issued guidelines were modified from WHO and international standards, but they were not tailored to local requirements [193].

## 7. Conclusions and Recommendation

The occurrence of As in food and water is a major health concern and substantial steps are required to overcome such challenging issues to obtain a healthy life and control health hazards associated with As and its various species. There are different species of As; some of them are highly toxic and some are nontoxic in nature, such as arsenate, arsenite, dimethylarsinate, methylarsonite, arsenobetaine, trimethylarsonio propionate, trimethylarsine oxide, etc. Certain effects of arsenic have been seen in human health but of more concern is maternal, fetal, and child health. Particularly, children up to five years of age are more vulnerable to As-sensitive effects due to more application of baby foods. Several As-associated complications in newborns and children have been reported, including spina bifida, deficits in memory and attention, stillbirth, slow fetal growth, low birth weight, delays in cognitive function, and neonatal and infant mortality. As exposure effects such as immunosuppression, anemia, GDM, breast cancer, cervical cancer, and early menopause have been seen in various women. It is the need of the hour to pay more attention to the mineral contents of the land used for the cultivation of cereals to avoid toxic arsenic exposure through plant sources. Specific guidelines on the acceptable concentrations of As in water and foods, particularly in baby foods, should be mentioned by different countries with toxic levels of different arsenic species. Furthermore, efforts are required to know the effects of food processing and the addition of different food processing aids or chemicals in different As species present in foods. The development and validation of analytical methods to measure the toxic levels of various As contaminants in different food products, particularly grains and grain-based products and rice and rice-based products, is the basic need of the entire world to conquer current As health hazards. Study of the toxicities of As in humans should be explored through various classifications on the basis of gender, age, and demographics. The scientific data of present information, particularly in the Middle East, provides novel insights on As toxic exposure and adverse health effects in different groups of people and levels of As in different foods and water. Furthermore, research and data collection are required to get present levels of As in different foods, including ready-to-eat food, baby food, and other exported food from different countries, water, vegetables, and fruits growing in As-rich land.

## Figures and Tables

**Table 1:** Different species of arsenic and their distribution in various foods with toxicities.

Different Species of Arsenic	Abbreviation	Distribution	References
Arsenocholine	AC	Arsenic species generally found in seafood and oxidized to arsenobetaine in a biological system.	[6]
In organic arsenic	iAs	Found in most foods and its presence in water is in low amounts.	[7]
Arsenite	As (III)	It is highly toxic in nature but present in lesser amounts in most foods.	[7]
Arsenate	As (V)	It is highly toxic in nature but present in lesser amounts in most foods and water.	[8,9]
Dimethylarsinate	DMA	Found in seafood and terrestrial foods and is a urine metabolite of iAs arsenosugars.	[10]
Dimethylarsinite	DMA (III)	It cannot be detected in food samples. It is a metabolite of iAs and can be seen in human urine samples but is highly toxic in nature.	[11]
Methylarsonate	MA	Found in seafood and terrestrial foods in very low amounts and is a metabolite of iAs that can be seen in urine.	[12]
Methylarsonite	MA (III)	It cannot be detected in food samples. It is a metabolite of iAs that can be seen in human urine samples but it is a toxic metabolite.	[13]
Arsenobetaine Arsenosugar	AB	It is a major arsenic species and commonly found in seafood but is non-toxic in nature.	[7]
Trimethylarsonio propionate	TMAP	Present in most foods. It is one of the major arsenic species.	[14]
Trimethylarsine oxide	TMAO	It is generally found in seafood and distributed in small amounts.	[11]

**Table 2:** Various concentrations of arsenic in the water of different Middle East Countries.

Study Area	Source	As Concentration Range (μg/L)	References
Kuwait	Marine water and sediment	0–43	[30]
Oman Sea	Fish species	0.74 ± 0.37 to 3.30 ± 1.39 μg/g	[31]
Kuwait	Kuwait Bay and the Open Sea		[32]
District of Baniyas, Abu Dhabi/UAE	Household drinking water	Max. 95.04	[29]
		Avg. 12.75	
		Min. 8.9	
Chabahar Bay/Oman	Surface sediments	5 and 22 ppm	[33]
Saudi Arabia/Al-Kharj agricultural region	Boreholes	Max.122	[35]
		Avg.31.18	
		Min. 2	
Chabahar Bay/Oman	Surface sediments	8.67–21 ppm	[42]
Al Jabal Al Akhdar/Oman.	Aflaj		[34]
Saudi Arabia/Gulf of Aqaba	Groundwater samples (wells)	Max. 2.2	[38]
		Avg. 0.63	
		Min. < 0.1	
Saudi Arabian Gulf coast, Tarut	Sediments, seawater, and gastropod and bivalve specimens	53–342 sediments	[43]
		8.55–14.88 seawaters	
		16.3–38.1 Molluscs	
Saudi Arabia/Arabian Gulf	Surface sediments	148	[44]
Al Munawarah area, Al Madinah/Saudi Arabia	Groundwater samples (wells)	<detection limit	[45]
Saudi Arabia/Gulf of Aqaba	Surface seawater and sediments	12.2–15.1 sediments	[40]
		0.46–1.55 Sea water samples	
Bahrain	Water fishing areas	0.85–2.75	[46]
Gulf and Oman’s Gulf	Marine biota (fish and various bivalves) and coastal sediments	Coastal Sediments	[41]
		Qatar 1.0–6.3	
		UAE 0.7–9.6 Bahrain 3.16–6.88	
		Oman 0.74–5.01	
		Molluscs	
		Gulf of Oman and Arabian Gulf 11.1–156	
Kuwait	Wells	<detection limit, 0.005 mg/L	[47]
Kuwait coast	Seawater, microplankton, shrimp, mollusk, fish	0.01–0.06 Water	[39]
		0.01–0.10 Particulate matter	
		0.01–0.04 Phytoplankton	
		0.08–0.42 Shrimp	
		0.15–0.43 Mollusc	
		0.21–2.10 Fin Fish	

**Table 3:** Total inorganic arsenic contamination in different foods available in our daily life with their toxic limits and detected values.

Different Dietary Products	Toxic Limits	Food Source	Total as Detected	References
Rice species	(0.10 mg/kg–0.30 mg/kg)	American Rice	0.25 mg/kg	[51]
		Thai rice	0.2 mg/kg	
		Pakistani rice	0.14 mg/kg	
		Indian rice	0.103 mg/kg	
		Egyptian rice	0.097 mg/kg	
Infant cereals	(0.1 mg/kg)	Rice	0.160 mg/day	[33,34,52,53]
		Milk powder	20 μg/kg	
		Fruit and vegetable mix	49 μg/kg	
		Mixed cereals	55 μg/kg–158 μg/kg	
Beans			Cowpea/Black-eyed pea	[54]
Meat products	Liver; 0.20 mg/kg Kidney; 0.50 mg/kg Fish; 4.0 mg/kg	Canned Meat	0.002 mg/kg	[55,56]
		Canned Fish	0.857 mg/kg	
Sea food/fish	6.0 μg/g	Shrimps Clams and pearl oyster Mollusk shells Saudi Arabia	0.19–0.53 μg/g	[57,58]
			11–134 mg/kg	
			16.3–38.1 mg/g	
Dairy products		Milk	0.0002–0.05 mg/kg	[59]
Vegetables	0.1 mg/kg 1.0 mg/kg (China)	Fresh vegetables	1.93–5.73 mg/kg	[52,53,60,61]
		Canned vegetables	2.50–5.10 mg/kg	
		Mushroom (Bangladesh)	0.51 mg/kg	
Dates	0.1 mg/kg	Eklas (Al-Hasaa)	0.584 mg/kg	[62]
		Barny (Al-adina)	0.078 mg/kg	
		Sakay mabroum (Al-Karj),	0.095 mg/kg	
		Sakay Nomal (Al-Karj),	0.109 mg/kg	
		Kadary (Al-Qaseem)	0.121 mg/kg	
Juices and beverages	0.01 mg/L	Canned beverages Non-canned beverages Juices Orange juice	0.003–0.161 mg/L	[63,64,65]
			0.002–0.261 mg/L	
			3.76 μg/kg	
			(1.137–18.36)	
			2.01–2.56 mg/kg	
Honey	15 μg/kg	Albaha Saudi Arabia	0.02–533.7 μg/kg	[66]
Tea and coffee		Organic oolong tea (China)	0.06–1.12 μg/L	[67]
		Granulated black tea (India)	2.680 mg/kg	
House hold water	10 μg/L	Commercial botteled Riyadh	0.574 ± 0.748 μg/L	[68,69]
Groundwater samples		Madina Al Munawwarah	12.0–29.0 μg/L	

**Table 4:** Various arsenic species and associated health hazards.

Arsenic Chemical Forms	Health Effects	References
Inorganic arsenic (As^III^ and As^V^)	Cancer	[100]
	Chronic diseases	[101,102]
	Mutation	[103]
	DNA damage	[104]
	Mitochondrial dysfunction	[105]
	Reduces bone mineralization	[106]
	Hyperglycemia	[107]
	Lipid disorders	[108]
	Coronary heart disease	[109]
	Liver toxicity	[110]
	Hypertension	[111]
	Genotoxicity	[112]
Arsenite (As^III^)	Cancer	[113]
	Fatty liver	[114]
	Hepatotoxicity	[115]
Arsenic trioxide	Breast cancer	[116]

**Table 5:** Direct effects of arsenic species and their metabolites with respective mechanisms in different chronic diseases.

Arsenic Species	Direct Toxic Effect and Target Organ Damage (TOD)	Molecular Mechanisms	References
Inorganic arsenic in drinking water and rice	Skin cancer	Differentiation and generation of cancer stem cells	[122,123]
	Coronary artery disease and cardiac muscle damage	Cardiac tissue hypoxia and inflammation	[124]
	Diabetes and insulin resistance	Inhibition of glycolysis, Krebs’s cycle, and ATP synthesis	[125,126]
	Acute Kidney Injury (AKI)	Kidney injury molecule-1 (KIM1)	[127]
	Chronic kidney disease (CKD)	Decreased glomerular filtration rate	[128]
Arsenite (As^III^)	Insulin resistance and metabolic syndrome	Diminished translocation of GLUT4	[129]
MMA (Monomethylarsonic acid)	Breast cancer	Endocrine disruptor	[130]
	Lung cancer	DNA damage	[104,131]
	Kidney cancer	DNA damage	[131]

**Table 6:** Arsenic levels in various foods in different countries.

Country Name	Rice mg/kg	Fish/Seafood mg/kg	Vegetables mg/kg	References
Bangladesh	0.021–0.66	1.80 ± 1.01	0.17	[189,190,191]
India	0.291–1.411	0.01–0.63	0.312–1.464	
Brazil	0.212	0.233	0.27	
China	0.186	0.0377	-	

## Data Availability

Not applicable.
